# Pre-exposure prophylaxis (PrEP) in primary health care: A qualitative study of users’ therapeutic itineraries in the Brazilian Amazon

**DOI:** 10.1186/s12889-026-28273-5

**Published:** 2026-07-22

**Authors:** Patrícia Saraiva Araújo, Diego Rafael Lima Batista, Priscila Saraiva Becil, Aline Cristiane Corte Alencar, Vinícius Azevedo Machado, Stefanie Costa Pinto Lopes, Marcus Vinícius Guimarães Lacerda, Djane Clarys Baia-da-Silva

**Affiliations:** 1 Instituto Leônidas & Maria Deane, Fiocruz Amazônia, Manaus, Brazil; 2 Fundação de Medicina Tropical Dr. Heitor Vieira Dourado, Manaus, Brazil; 3 Universidade do Estado do Amazonas, Manaus, Brazil; 4 Universidade Nilton Lins, Manaus, Brazil

**Keywords:** HIV, Pre-exposure prophylaxis, Treatment itinerary, Primary health care, Brazilian Amazon

## Abstract

**Background:**

Pre-exposure prophylaxis (PrEP) for HIV is available free of charge through the Brazilian Unified Health System, yet little is known about users’ experiences in primary health care in the Brazilian Amazon. This study describes and analyzes the therapeutic itineraries (TI) of PrEP users attending a Family Health Unit (FHU) in Manaus, Amazonas.

**Methods:**

This is a qualitative, exploratory, and descriptive study based on in-depth interviews conducted between July and September 2023. Twenty HIV-negative adults (cisgender men and women, and one transgender man) using PrEP at FHU were included. Data were analyzed using MAXQDA. TI were interpreted using Arthur Kleinman’s Health Care System model as an analytical framework.

**Results:**

Participants learned about PrEP through media, social networks, partners, friends, and previous experiences with post-exposure prophylaxis (PEP). Information acquired through interpersonal relationships was frequently legitimized through professional care, where testing, counselling, and access to PrEP transformed awareness into prevention practices. Continuity of care was influenced by distance, transportation costs, work schedules, stigma, and concerns about confidentiality. Primary care services were often perceived as more accessible and less stigmatizing than specialized HIV services. Interruptions and resumptions of PrEP use reflected ongoing reassessments of risk and prevention needs, while trust and supportive care relationships facilitated continuity.

**Conclusion:**

TI revealed that PrEP use is a dynamic process shaped by interactions among social networks, institutional practices, stigma, and everyday prevention strategies. Strengthening decentralized, flexible, and privacy-protective primary health care services may reduce barriers and support equitable HIV prevention in the Brazilian Amazon.

**Supplementary Information:**

The online version contains supplementary material available at https://doi.org/10.1186/s12889-026-28273-5.

## Introduction

In 2024, approximately 40.8 million people were living with HIV worldwide, with an estimated 630,000 deaths from AIDS-related illnesses [1], while Brazil recorded 36,055 new cases of AIDS [2]. Detection rates in the country’s northern region, especially in Amazonas, remain consistently above the national average. The state recorded 25.4 cases per 100,000 inhabitants in 2023, compared to the national rate of 11.8 [2]. Since 2017, pre-exposure prophylaxis (PrEP) has been available free of charge through the Brazilian Unified Health System (SUS) and has proven highly effective when correctly administered [3, 4]. However, the effectiveness of PrEP as a public health strategy depends not only on availability but also on timely access, continuity of use, and sustained engagement with health services [5].

In addition to its proven effectiveness in public health, PrEP has become a fundamental element in HIV prevention strategies within health systems, especially the Brazilian SUS [6–8]. Expanding access to PrEP through primary health care (PHC) contributes not only to expanding the supply of preventive services but also to strengthening longitudinal care, reducing the stigma associated with specialized HIV services, and fostering more comprehensive and community-based approaches to sexual health [9–11]. Thus, the therapeutic itinerary (TI) framework is relevant for analyzing how individuals move between different care sectors, mobilize social and institutional resources, and encounter barriers and facilitators throughout their prevention trajectories [12–14]. By considering everyday experiences, social relationships, and interactions with health services, TI broadens understanding of PrEP use beyond strict biomedical perspectives or those focused on individual adherence [15–21]. Studies of TI in PHC, particularly in Family Health Units (FHUs) in the Amazon region, remain rare. Understanding how PHC influences access to, continuity of, and return to PrEP use is essential, given its central importance in expanding prevention coverage and reducing stigma through the decentralization of HIV care.

This study describes and analyzes in depth TI of PrEP users attending a Family Health Unit (FHU) in Manaus, from first contact through maintenance of use, with emphasis on access issues, the challenges faced, support dynamics, and strategies to ensure continuity. Attention is given to issues related to access, continuity of care, support networks, barriers, and everyday strategies adopted to sustain prevention practices. Unlike much of the existing literature, which has predominantly focused on PrEP delivery among men who have sex with men (MSM) in specialized services [22–24]. In this study, Kleinman’s proposed Health System model is used as an interpretative framework to understand the participants’ experiences, without the intention of exhaustively describing all sectors of the model. Shifting the focus from individual adherence to a health service-mediated perspective, the study broadens understanding of how to effectively deliver PrEP in decentralized contexts, where resource limitations pose challenges.

## Methods

### Ethical aspects

This study was conducted in accordance with the principles established in the Declaration of Helsinki [25] and reported in accordance with the consolidated COREQ criteria for qualitative studies [26] (see Supplementary Material 1). The research was approved by the Research Ethics Committee of Fundação de Medicina Tropical Doutor Heitor Vieira Dourado (FMT-HVD) under opinion CAAE 56334222.00000.0005. All participants signed the informed consent form before participating. The audio files and transcripts were duly anonymized, stored on password-protected devices, and kept under restricted access to the research team.

### Study design and study location

This qualitative, exploratory study investigates the TI associated with PrEP use in a FHU in Manaus, Amazonas. The TI framework adopted in this study is grounded in Arthur Kleinman’s Health Care System model [27]. Rather than treating the popular, professional, and traditional sectors as fixed or isolated categories, Kleinman’s Health System model was adopted as an interpretative framework to understand how participants moved through interconnected fields of care along their TI. These itineraries encompass not only the routes through health services, but also the dynamic processes in which individuals interpret risks, mobilize social and institutional resources, negotiate prevention strategies, and reorganize daily care practices over time [27–30]. This perspective guided the analysis of how social relations, institutional practices, stigma, territorial barriers, and everyday experiences influence therapeutic decisions, continuity of care, and PrEP use.

Considering that the analysis relied exclusively on interviews with PrEP users, the study does not intend to exhaustively reconstruct all the care sectors outlined by Kleinman. Its focus is on exploring how these sectors were perceived, experienced, and negotiated from the users’ perspective throughout their therapeutic itineraries.

In-depth interviews (IDIs) were conducted between July and September 2023 and targeted PrEP users served at Dr. Raimundo Franco de Sá FHU (RFS-FHU), the first PHC unit in Manaus, Amazonas, to offer PrEP.

Manaus is the capital of the state of Amazonas (population approximately 2,000,000 inhabitants) and reports the highest HIV notification rates in Brazil [2]. In 2023, PrEP was offered at 10 health units in the urban area, including one at FMT-HVD, a referral unit for HIV/AIDS in the state of Amazonas, and the others distributed across the FHUs.

RFS-FHU provides PrEP initiation and follow-up delivered by trained physicians and nurses, in accordance with local protocols. This includes periodic clinical consultations and HIV/STI testing, as well as routine laboratory monitoring (e.g., renal and hepatic function). PrEP was offered to a heterogeneous population, including heterosexual women in serodifferent relationships and transgender people.

### Participants and recruitment

Eligible participants were adults aged ≥ 18 years, self-reported as HIV-negative, enrolled in PrEP follow-up at the RFS-FHU, and using PrEP at the time of this research. There were no restrictions regarding sexual orientation or sex assigned at birth to encompass a broader and more diverse group of participants. Participants were selected based on predefined inclusion criteria and identified using the FHU registry. Individuals were contacted by phone and invited to participate. Recruitment continued until thematic saturation was reached. The definition of thematic saturation was reached by consensus among the researchers, based on code repetition, the recurrence of attributed meanings, and the absence of new analytical findings.

To characterize the profile, we collected basic sociodemographic information, including each participant’s residential area (Table 1).

**Table 1:** Characteristics of the interviewees

Interviewees	Age	Education in years	Profession	Gender identity	Sexual orientation	Approximate time (in months) of use of the PrEP
01	27	≥ 15	Nutritionist	Cis man	Homosexual	5
02	25	≥ 15	Advertising	Transgender man	Bisexual	24
03	25	≥ 15	University student	Cis man	Homosexual	30
04*	48	≥ 15	Technician	Cis woman	Heterosexual	28
05*	39	11	Home carer	Cis woman	Heterosexual	36
06*	37	11	Freelance	Cis woman	Heterosexual	6
07	39	≥ 15	Artistic producer	Cis man	Homosexual	21
08	26	11	Freelance	Cis man	Homosexual	17
09	30	≥ 15	University student	Cis man	Homosexual	40
10*	37	11	Home carer	Cis woman	Heterosexual	12
11*	39	11	Freelance	Cis woman	Heterosexual	24
12	22	≥ 15	University student	Cis man	Homosexual	48
13	27	≥ 15	Sales agent	Cis man	Homosexual	30
14*	26	≥ 15	Doctor	Cis man	Homosexual	18
15	31	≥ 15	University student	Cis man	Bisexual	6
16*	39	≥ 15	Doctor	Cis man	Homosexual	11
17	25	≥ 15	Advertising	Cis man	Bisexual	12
18	23	≥ 15	University student	Cis woman	Bisexual	24
19*	47	≥ 15	Nursing technique	Cis woman	Heterosexual	38
20*	40	11	Domestic worker	Cis woman	Heterosexual	20

*Participants in serodifferent partnership

### Data collection (in-depth interviews)

We developed a semi-structured interview guide (SSIG) to investigate: (1) how participants first came into contact with information about PrEP, (2) the pathways they followed to obtain PrEP through the FHU, (3) their experiences interacting with services and professionals involved, and (4) strategies and difficulties faced in maintaining PrEP use in daily life (see Supplementary Material 2). The SSIG underwent a preliminary evaluation with four volunteers. They were not included in the final analysis, allowing for minor adjustments before fieldwork began.

Interviews were conducted by trained researchers with experience in qualitative methods who were not affiliated with RFS-FHU and had no prior relationship with the participants. Interviews were conducted in private settings selected for this purpose, with audio recording authorized. Each interview lasted approximately 45 min. The entire procedure was conducted in Portuguese, with full transcription of the speech and sFHUequent anonymization of the data. Selected quotations, used as illustrative examples, were translated into English and reviewed by another team member to ensure semantic equivalence.

### Data analysis

The interview transcripts were entirely produced and organized in MAXQDA20. Data analysis followed an iterative, team-based approach combining deductive and inductive coding. We developed an analytical code based on the TI phases. This initial structure was expanded through open coding, incorporating meanings and themes that emerged directly from the participants’ accounts.

Three researchers (PA, PB, DB) independently coded an initial codebook of transcripts; they then met to compare results, align definitions, and refine codes (Supplementary Material 3). The improved version of these codes was then applied to the complete dataset and updated as needed. Regular meetings facilitated discussion of disagreements and consolidation of collective interpretations, thereby promoting greater rigor.

### Research team and reflexivity

The research team comprised experienced qualitative researchers, all with backgrounds in infectious diseases and public health. All members possessed specific training in qualitative methods. Critical reflection on their own assumptions was systematically addressed through regular meetings throughout the data collection and analysis process, with the aim to reduce the influence of perspectives on results interpretation.

## Results

**Fig. 1:**
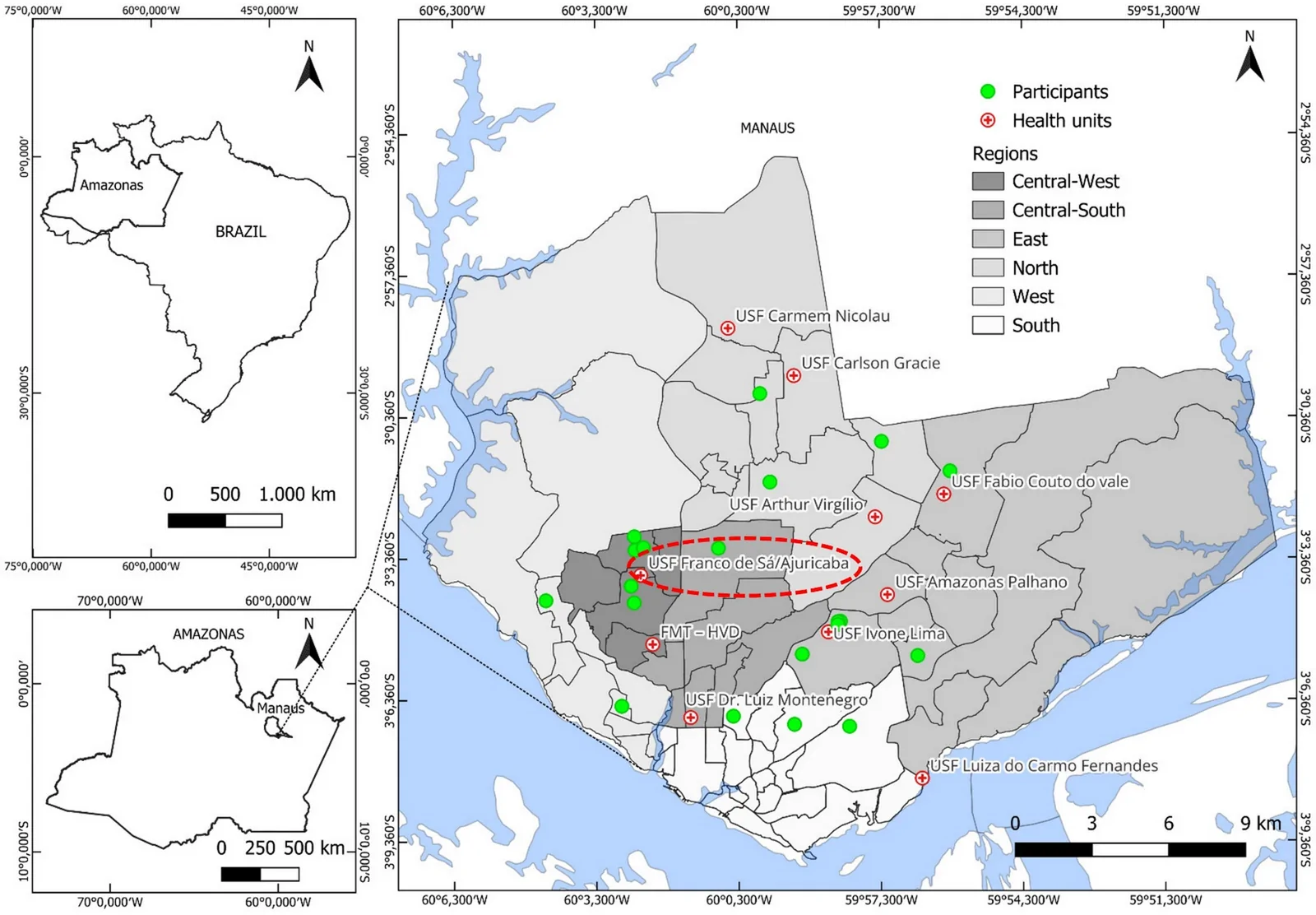
Location of PrEP service units at the study’s time and participants’ residence

Thirty-one people were invited, but only twenty were included. The mean age was 32 years, and the duration of PrEP use ranged from 6 months to 4 years. The participants comprised cisgender men (*n* = 11), cisgender women (*n* = 8), and one transgender man (*n* = 1). Regarding sexual orientations, there were gay/MSM (*n* = 9), bisexual cisgender men (*n* = 2), and heterosexual women in serodiscordant partnerships (*n* = 7). The educational level was high across much of the sample, with 11 participants having completed higher education (Table 1). The concentration of participants with higher levels of education suggests that structural inequalities limit access to PrEP, especially among the most socioeconomically vulnerable, who may face even greater obstacles. Most reside in neighborhoods far from the RFS-FHU (*n* = 12) (Fig. 1).

Results are presented according to Kleinman’s Health Care System model. Participants’ narratives primarily reflected the popular and professional sectors, whereas elements related to the folk sector emerged less frequently. In addition, facilitators, barriers, and patterns related to PrEP use emerged across multiple care sectors. Supplementary Material 4 presents additional interview excerpts organized according to Kleinman’s Health Care System model.

### Popular sector and social networksx’

The popular sector encompasses everyday social relationships and experiences that shape how participants learn about PrEP, interpret it, and engage with its use. Knowledge about PrEP emerged through relational and social networks, rather than relying exclusively on institutional communication channels. Friends, partners, prior experiences with PEP, and exposure to different media influenced participants’ perceptions of HIV prevention and drove their pursuit of PrEP. These findings suggest that access to PrEP is socially mediated and embedded in daily relationships based on trust, perceived vulnerability, and shared experiences.

#### a) Initial knowledge about PrEP

Participants mentioned various ways in which they first met PrEP, including television reports, social media, peer support networks, and interactions with partners and healthcare professionals. Knowledge about PrEP arose primarily through these social and relational networks, rather than through institutional communication channels, highlighting how preventive practices are socially mediated in participants’ daily lives. Informal exchanges with friends, partners, and healthcare professionals frequently sparked curiosity about PrEP and encouraged the search for preventive services.

*“The first time I heard it was on the news. I was watching a report*,* that’s when it appeared.”* (Participant 10 – cis woman – heterosexual – serodifferent partner).

*“I have two friends who were taking it and then I became interested. I went to find out how it worked (…) I talked to the nurse*,* and she let me take it due to the circumstances.”* (Participant 18 – cis woman – bisexual).

Relationships with serodiscordant partners also stood out as important motivators for initiating PrEP use, especially because they increased awareness about HIV prevention and reinforced feelings of vulnerability, protection, and shared responsibility within intimate relationships.

These findings indicate that the decision to initiate PrEP was not experienced solely as an individual biomedical choice, but as part of broader information technologies shaped by interactions between social relationships, perceived HIV risk, and institutional care opportunities. The circulation of information between interpersonal networks and healthcare professionals demonstrates how different segments of the care system dynamically interact throughout the participants’ prevention trajectories.

#### b) Personal motivators for starting and adhering to PrEP

Decisions to initiate and maintain PrEP were influenced not only by biomedical factors but also by emotional experiences of fear, protection, autonomy, and trust in prevention strategies. Participants frequently reported PrEP as part of a larger set of efforts to cope with vulnerability and regain control over sexual health and intimate relationships.

Previous situations perceived as risky, especially unprotected sex or experiences with PEP, increased the perception of vulnerability to HIV and served as important transitional moments for initiating PrEP.

*“I had sex with a partner and had sex without a condom. So*,* I ran to the hospital to use PEP. The doctor told me that after I finished the PEP treatment*,* I should look for PrEP”* (Participant 8 - Cis man - homosexual).

Fear of HIV infection and the desire for protection stood out as central motivations for initiating prophylaxis, especially among participants in serodiscordant relationships or who reported casual or multiple sexual partners.

“*I accepted it out of desperation*,* out of fear of contracting HIV. Fear of taking a test and finding out that I had contracted the virus (…) I just wanted to prevent it. I just wanted to have the minimum risk of contracting it*” (Participant 6 – cis woman – heterosexual – serodifferent partner).

Participants also linked the use of PrEP to an increased sense of security, confidence, and autonomy in conducting sexual relations and in prevention practices.

*“I was getting involved with people. I didn’t know very well or who were just flings (…) I decided to take it because at least I will have more security.”* (Participant 18 – cis woman – bisexual).

Confidence in the effectiveness of PrEP was strengthened through personal experiences and by comparison with previous situations of HIV exposure.

*(…) I trust it a lot. Because I’ve already had three experiences with three people I was in a relationship with*,* and they got infected with HIV and I didn’t.”* (Participant 12 – cis-homosexual man).

#### c) Understanding prophylaxis

Participants generally demonstrated adequate knowledge regarding the main purpose and use of PrEP, recognizing it as a daily medication intended to prevent HIV infection. Understanding of PrEP was commonly associated with notions of protection, effectiveness, and continuity of use. However, differences in depth of knowledge emerged among participants, particularly among some married women, whose narratives suggested less familiarity with the broader scope of preventive care and PrEP management.

*“It protects against HIV (…) it is 99% effective and cannot fail. It is used daily”* (Participant 06 – cis woman – heterosexual).

Participants also highlighted that PrEP does not prevent other sexually transmitted infections (STIs), reinforcing the perception that additional preventive measures are still necessary.

*“I am fully aware that PrEP protects against HIV; it does not protect against other diseases”* (Participant 13 – cis man – bisexual).

### Professional sector and institutional care

This sector encompasses formal health services, infrastructure, professional support, access to care, and follow-up practices related to PrEP use. Participants’ experiences showed that the organization of health services, territorial accessibility, resource availability, and relationships with professionals directly influenced continuity of care and adherence to PrEP. PHC was frequently considered a more accessible and less stigmatizing environment for HIV prevention, despite the persistence of significant structural and logistical barriers.

#### a) Access and resources needed

Access to PrEP in PHC was heavily affected by territorial and organizational factors, including distance to services, transportation costs, waiting times, and compatibility with work routines. Participants’ narratives indicated that entry into and continuity of PrEP care depended not only on individual motivation but also on the availability of financial, social, and institutional resources that facilitated navigation within the health system.

Informal guidance from health professionals and social contacts often facilitates access, helping participants identify available services and initiate PrEP use. Geographical distance, transportation costs, and scheduling requirements emerged as significant barriers to continuity of care, especially among participants experiencing economic vulnerability.

*“It’s far from my house. I go by public transportation. It takes about 25 minutes to get there. When there aren’t many people*,* I can get everything done in 1 to 1.5 hours and then return home. There’s a PrEP room where I also wait until I’m seen*,* and there are many people. It takes a while*,* but it’s worth it”* (Participant 15 – Cis man – bisexual).

*“I go out during my lunch break (…) It’s far. (…) I always take Uber.(…) If we go by bus*,* we must pay two or three fares. So*,* it becomes more expensive”* (Participant 11 – Cis woman – Heterosexual - Serodifferent partner).

For some participants, economic difficulties directly impacted on their ability to attend follow-up appointments and to purchase medications.

*“I’m currently unemployed; there are days when I don’t even have money to eat*,* let alone pay for the bus fare to get PrEP”* (Participant 10 – Cis woman – Heterosexual - Serodifferent partner).

#### b) Health care

Participants frequently contrasted PHC services with specialized HIV centers, perceiving primary care as more discreet, accessible, and capable of ensuring continuity of care. Thus, the evaluation of healthcare experiences went beyond the simple availability of services, being strongly marked by perceptions of stigma, trust in professionals, and the ease with which participants could integrate care into their daily routines.

A recurring theme was the perception that FHUs provide greater privacy and less exposure to the stigma associated with HIV compared to specialized services. By addressing a diversity of health needs, FHUs made participants feel less vulnerable to social judgments and unwanted assumptions about their serological status.

“*If someone sees me leaving the referral unit with a bunch of vials*,* what will they think? (…) But if someone sees me leaving the FHU with many vials*,* it’s less uncomfortable because they treat everything there.”* (Participant 14 – cis man – homosexual).

Participants also highlighted the practical advantages of decentralized care. Compared with specialized services, FHUs were often seen as more accessible and efficient, facilitating access to PrEP and reducing scheduling and follow-up barriers.

*“I came Tropical Medicine Hospital*,* Reference Unit Referral unit for HIV treatment*,* and I found it more difficult because I had to make an appointment with the infectious disease specialist. It was scheduled*,* but I couldn’t make it that day. There (FHU)*,* I arrived*,* went*,* and told the nurse that I wanted to take PrEP*,* and I was seen right away and got the medication the same day”* (Participant 0 – Cis man – Homosexual).

The relationships established with healthcare professionals played a central role in participants’ engagement with PrEP. Respectful communication, active listening, and a non-judgmental attitude helped build trust and encouraged continued use of prevention services. On the other hand, negative interactions generate discomfort and could discourage seeking care.

*“There is a professional there who is very good. He takes good care of us. We feel comfortable talking about things. He is not in a bad mood. As it happened*,* I arrived at a health center with secretions from my penis and the professional was super rude to me.”* (Participant 07 - Cis man - homosexual).

Participants also valued the organizational structure of the FHUs, highlighting the availability of health professionals, laboratory services, pharmacies, and other resources that contributed to effective monitoring and continuity of care.

*“I think the infrastructure is cool. There are laboratories there*,* there are professionals available*,* there are computers*,* a pharmacy. I think it’s good” (Participant 3 – Cis man – homosexual)*.

#### c) Service and follow-up time

Participants described the follow-up process as organized and accessible, although they noted expectations and bureaucratic requirements for consultations and laboratory monitoring. Regular consultations, rapid tests, medication dispensing, and periodic clinical visits were perceived as essential elements for continuity of care, contributing to a sense of security and peace of mind regarding HIV prevention.

Although some participants reported waiting times of 20 min to 1 h for appointments, these intervals were generally considered acceptable given the demand for services. They also highlighted the availability of rapid tests, constant monitoring, and easy access to healthcare professionals as positive aspects of PrEP follow-up.

*“I get a ride with my father on the way there (…) The most I’ve ever spent at the FHU was an hour. But because there are people in front of me*,* the screening itself*,* the exam*,* everything is rapid”* (Participant 2 – Trans man – bisexual).

At the same time, the need to schedule appointments, undergo laboratory tests, and return to receive the results was sometimes perceived as bureaucratic and time-consuming.

*“We do the rapid tests. We go to the doctor*,* to the nurse. He asks a lot of questions… And he gives us the medicine*,* but we must go back to do the tests. Which is the complete blood count*,* HIV*,* syphilis*,* hepatitis*,* kidney function… I think it’s great!”* (Participant 8 – Cis man – homosexual).

#### d) Consultation and receipt of medication and daily use

Participants reported that maintaining PrEP use required ongoing organization of appointments, laboratory tests, medication pick-up, and daily adherence practices. TI involved constant negotiation between healthcare demands, work routines, mobility, and daily responsibilities, requiring users to develop individualized strategies to sustain prevention over time. Participants frequently adjusted medical appointments and follow-up visits to accommodate work schedules and other daily commitments, highlighting the active management necessary to ensure continuity of care.

*“I usually go in the morning for a consultation or exam. Then I go to work. (…) When they schedule an afternoon appointment (consultation or exam)*,* I ask to leave work. I go and come back (…) it’s easy”* (Participant 02 – trans man - bisexual).

The daily use of medication was incorporated into personal routines through reminders, organizational strategies, and adaptations to social activities, seeking to ensure adherence to treatment.

*“I take it every night. I prefer to take it at night (…) at eight-thirty o`clock. If I go out somewhere*,* I put a pill in a tiny container*,* and it goes with me in my bag. When it’s eight thirty*,* my cell phone wakes up wherever I am. It’s time to take my medicine.”* (Participant 4 – Cis woman – Heterosexual - Serodifferent partner).

#### e) Criticism and suggestions

Participants highlighted organizational and communication difficulties that impacted their experiences in monitoring PrEP in primary health care services. Frequent visits to health units for consultations, laboratory tests, and medication pick-up were considered a burden, especially when combined with work routines, transportation difficulties, and waiting times.

*“(…) we need to go and schedule an appointment. To schedule the exam*,* go on the day of the exam*,* take the exam*,* and schedule a new one. Every 4 months I must go there 3 times. If it were just one day*,* it would be good” (Participant 3 – Cis man – Homosexual)*.

Participants also highlighted the importance of communication and organization in healthcare services. The lack of information about how services operated and the uncertainty during waiting periods generated dissatisfaction and fostered a perception of disorganization.

Concerns arose regarding confidentiality and stigma, especially concerning the visibility of PrEP medication in public spaces. Participants requested more discreet packaging to avoid embarrassing situations.

*“When you get the bottles*,* you must carry them in your hand… Or you put them in your pocket… Or if you have a bag… But if we don’t carry a bag… there are several rather large bottles. If there was a small bag that wasn’t transparent*,* it would be more comfortable for some. Because people stare”* (Participant 08 – Cis man - homosexual).

### Elements related to the folk sector

Elements related to the folk sector appeared only occasionally in the participants’ narratives and, consequently, received a more limited analysis in comparison to the popular and professional sectors, mainly related to stigma, moral judgments, and informal beliefs about HIV and PrEP. The accounts revealed social meanings that associate PrEP use with HIV infection, promiscuity, or socially disapproved sexual behaviors, demonstrating how these informal beliefs influence the declaration of use, social interactions, and the continuity of preventive practices. Although less evident than in the popular and professional sectors, these testimonies show how social perceptions and moral judgments structure the participants’ experiences with PrEP and their therapeutic journeys.

#### a) Prejudice and discrimination

Participants described experiences in which PrEP use was socially associated with promiscuity, HIV infection, or disapproved sexual behaviors. These perceptions generated embarrassment, fear of judgment, and led to the concealment of PrEP use within the family and on social media, affecting how they managed prevention practices in their daily lives.

*“I realize there is prejudice because people immediately think*,* ‘Look*,* this girl is for everyone!’. I think people see promiscuity. I think they see people as sluts”* (Participant 18 – cis woman – bisexual).

The fear of stigma also impacted decisions related to disclosing PrEP use, especially in situations involving serodiscordant relationships and family settings.

*“I don’t talk (…) I told my aunt*,* and she didn’t believe me. She said I was hiding HIV*,* that I had caught it from my husband. To protect him*,* I don’t tell anyone”* (Participant 20 – cis woman - heterosexual - Serodifferent partner).

Stigma acted as a significant social barrier, affecting both disclosure and communication and engagement with prevention practices. Moral judgments associated with HIV and sexuality shaped how participants managed the visibility, confidentiality, and social acceptance of PrEP use in their daily lives.

#### b) Communication and trust

Trust built within interpersonal relationships proved a key factor in disseminating information about PrEP and exchanging prevention-related experiences. Participants indicated that conversations about PrEP happened more often in contexts marked by emotional closeness, mutual trust, and supportive social connections. In these settings, users frequently took on active roles in spreading information, promoting preventive practices, and normalizing discussions about HIV prevention within their social networks.

*“(…) I’m very open; I told my parents about PrEP. They were shocked*,* but it was okay*,* I’m taking care of myself (…). Usually*,* when it’s a friend*,* as soon as I trust them*,* (…) who needs it*,* or who I have a good relationship with*,* I tell them”* (Participant 18 – cis-bisexual woman).

Participants also reported serving as informal sources of information and support for friends and acquaintances interested in PrEP, thereby promoting the dissemination of prevention knowledge beyond formal healthcare settings.

*“People get very curious when I talk (…) I talk excitedly (…) I talk about how it works*,* and I’ve already influenced a lot of people who I think need to use PrEP”* (Participant 9 – cis-homosexual man).

### Across sectors: Facilitators, barriers, and patterns of PrEP use

#### a) Facilitators

Participants highlighted several factors that facilitated both PrEP use and continuity of care. Trust built in relationships with partners, friends, family, and healthcare professionals promoted open dialogue about HIV prevention, reducing barriers to discussing PrEP. In this environment, prevention was more naturally incorporated into daily life, becoming less affected by stigma and social taboos.

Furthermore, participants associate the use of PrEP with stronger feelings of security, autonomy, and control over their own sexual health. For some, adopting PrEP brought greater confidence in managing HIV risk and making decisions about preventive practices.

*“I feel safer*,* mainly because I’m not in a relationship with just one person.* (Participant 1 – cis homosexual man)

Regular contact with health services proved to be another facilitating factor. Participants indicated that periodic follow-up appointments encouraged more comprehensive engagement with health care, going beyond HIV prevention and promoting greater attention to overall well-being.

*“The fact that I go to the FHU every three months as if it were my obligation makes me feel more confident about taking care of other things too. So*,* I started taking better care of myself*,* so I asked the nurse: I want to see the doctor*,* you know? Things that I didn’t usually do. So*,* I take advantage of it and do it”* (Participant 15 – cis-bisexual man).

The factors that facilitated the use of PrEP were strongly associated with supportive social relationships, a sense of protection, and consistent engagement with health services. These aspects contributed to the strengthening of preventive practices and the continuity of care over time.

#### b) Barriers

Participants identified stigma, fear of social judgment, and organizational demands related to clinical follow-up as significant obstacles to PrEP use and continuity. Difficulty discussing PrEP with family, friends, and intimate partners was frequently associated with fear of being perceived as HIV-positive, promiscuous, or involved in socially disapproved sexual behaviors. In certain situations, not disclosing PrEP use is also aimed to protect HIV-positive partners from stigma and unwanted exposure.

Participants reported that social perceptions related to PrEP use often associated it with promiscuity, sex work, or HIV infection, which caused embarrassment and encouraged the adoption of strategies to conceal its use.

*“They either think I’m a prostitute or that I have risky sexual exposure. That I’m promiscuous*,* a slut all the time*,* or that I live with someone who has HIV”* (Participant 14 – Cis man – homosexual).

In addition to the stigma, participants reported that consultations, laboratory tests, and follow-ups required significant planning and could interfere with professional responsibilities and daily routines, especially for those with little flexibility or long commutes to healthcare facilities.

*“I work independently; I work in production. I don’t always have demand. So*,* it’s more flexible. There are people who work all day*,* sometimes spending two hours on the bus”* (Participant 07 – Cis man – homosexual).

#### c) Decisions about PrEP use/discontinuation

Participants reported changes in prevention practices and condom use after starting PrEP, frequently associating prophylaxis with a greater sense of protection, autonomy, and sexual freedom. Simultaneously, decisions about continuing or discontinuing PrEP were impacted by varying perceptions of HIV infection risk, as well as factors such as relational dynamics, professional demands, and everyday situations.

Some participants highlighted greater flexibility in condom use after starting PrEP, especially in contexts marked by trust, emotional bonds, or a feeling of reduced vulnerability. Interruptions and resumptions of PrEP use were frequently described as integral parts of dynamic prevention strategies rather than as definitive abandonment of care. Changes in marital status, especially the beginning of monogamous relationships, often influenced risk perception and decisions to temporarily suspend prophylaxis.

*“I stopped for a few periods in these six months*,* but I always go back. I got into a monogamous relationship (…) I felt comfortable and trusted the person”* (Participant 3 – Cis man - homosexual).

Participants reported that workplace pressures, fear of stigma, and difficulties balancing healthcare demands with professional responsibilities compromised continuity of care and adherence to PrEP.

*“I stopped for a while (stopped using PrEP). (…)As a salesperson*,* I’m pressured to produce more (…) There came a point where the company was apprehensive*,* with a lot of people being fired. I didn’t want to leave my job to seek treatment because I was afraid of being frowned upon” (Participant 13 – Cis man – homosexual)*.

Throughout the participants’ TI, PrEP presented dynamic and non-linear trajectories, shaped by constant negotiations related to the perception of HIV risk, affective relationships, working conditions, stigma, and daily priorities. Maintaining PrEP use stood out as a flexible prevention strategy that was constantly reassessed and adjusted in response to social and contextual changes.

#### d) Behavior changes

Beyond the direct use of PrEP, participants identified significant behavioral and relational changes associated with this practice, such as strengthened self-care, greater attention to sexual health, and a more careful selection of partners. The experience with PrEP was often understood as a catalyst for reflection on preventive strategies and for increased awareness of personal vulnerability and appropriate forms of protection.

Additionally, some reported adopting a more cautious stance in their relationships and sexual decisions after starting to use prophylaxis, even when they felt more secure about HIV prevention.

*“I even started getting involved with people I already knew better*,* you know*,* with whom I already had a history*,* or something like that*,* but always with protection. But then I started to be more careful”* (Participant 18 – cis woman - bisexual).

The use of PrEP has promoted transformations that go beyond biomedical prevention, influencing daily care practices, risk perceptions, and the management of intimate relationships.

## Discussion

The TI reported by the participants did not develop linearly, nor were they restricted to formal health services. Information about PrEP and perceptions related to HIV risk frequently emerged through social networks, digital platforms, partners, friends, and previous experiences with infection prevention. These experiences stimulated contact with health services, where professional guidance, tests, and access to PrEP transformed informal knowledge into systematized prevention practices. The continuity of medication use depended on the participants’ ability to integrate the medication, follow-up appointments, and laboratory monitoring into their daily routine, while facing stigma, work demands, mobility limitations, and changes in affective relationships. Interruptions and resumptions in PrEP use did not necessarily indicate a departure from care; rather, they reflected ongoing reassessments of preventive needs and perceived vulnerability.

The results highlight the usefulness of Kleinman’s model as an interpretive tool for understanding how participants experienced and negotiated different dimensions of care throughout their therapeutic journeys. The popular and professional sectors are more widely represented, while the folk sector appeared only occasionally in the accounts. Information conveyed through social networks and interpersonal relationships frequently gained legitimacy and became effective in health services, while stigma, moral judgments, and concerns about confidentiality influenced how participants sought, experienced, and maintained care. Instead of functioning as isolated domains, popular, professional, and traditional sectors acted as interconnected fields, whose prevention practices were negotiated, adapted, interrupted, and reconfigured over time. This interpretation expands on findings from the Brazilian Amazon, which emphasized the importance of social networks and peer communication among key populations [22], by highlighting how these social processes continue to influence engagement, continuity, interruption, and resumption of prevention in the context of primary health care, including a more diverse group of participants, such as cisgender women in serodiscordant relationships and transgender people, in addition to the groups traditionally addressed in the literature.

In the general population, initial contact with PrEP rarely occurs solely through official campaigns or formal health initiatives. In fact, the decision to seek prophylaxis is often triggered by social dynamics and, frequently, by perceived or experienced risk events. Participants’ accounts reveal different entry points into this universe: social networks, friends, partners, bonds of trust, and even previous experiences with PEP. These findings align with previous studies, which indicate that both the discovery and adherence to PrEP are profoundly shaped by bonds of trust within peer networks and by presence in digital environments, especially among key populations [22–37]. A study conducted with MSM in Brazil, for example, demonstrated that most users learned about PrEP through peers and digital contacts, not through institutional campaigns [34]. Thus, restricting interventions to formal education may overlook critical steps that precede access to pharmaceuticals. Strategies such as community-targeted communication, accessible referral flows, guidance tools, and culturally situated materials prove to be as vital as the clinical consultation itself.

Experience with PEP also appears as an important gateway to PrEP, especially after recent HIV exposure events [38, 39]. This evidence reinforces two fundamental directions: integrating PrEP messaging into channels already present in users’ daily lives and articulating clear transition flows between PEP and PrEP, a practice internationally recognized for supporting continuity of care [40]. A recent WHO guideline identifies PEP care as a “teachable moment,” in which discussion of transitioning to PrEP with people who remain at risk should be prioritized [41, 42]. A longitudinal study shows that, after using PEP, the probability of initiating PrEP increases by more than fivefold, which justifies formalizing these referral flows [39].

The motivations for initiating and maintaining PrEP use go beyond reducing biomedical risk. Several studies show that those who use PrEP frequently associate its use with feelings of autonomy, security, and greater control over their sexual health, in addition to a decrease in HIV-related anxiety [43–47]. Cohort studies demonstrate parallel reductions in overall anxiety and HIV-related worry over 12 months among PrEP users [48]. These subjective benefits help explain why so many people continue to use PrEP despite logistical, social, or economic barriers, making it clear that adherence is not limited to a behavioral or biomedical issue. In line with qualitative research that emphasizes the symbolic and emotional dimensions of daily PrEP use [46–52], our findings reinforce the importance of policies and interventions that address these affective and social aspects, going beyond traditional clinical recommendations. PrEP, if understood as social technology, can act as a device that reconfigures the experience of risk. Users rework their forms of self-care, their sexual practices, and their ways of relating to others, producing new grammars of protection, trust, and autonomy. In this sense, it operates simultaneously on biological, social, and subjective levels, articulating knowledge, practices, and values, and demanding that public policies and health interventions recognize and incorporate this complexity, thereby overcoming strictly normative approaches or those centered on biomedical risk [53].

The professional sector played a crucial role in the trajectories of those who sought PrEP. Most participants reported that PHC services were more accessible, discreet, and less stigmatized than specialized HIV services. This is because PrEP was offered as part of routine care, without explicit signs of linkage to HIV treatment. However, practical issues always resurfaced in the accounts: distance to PHC, travel costs, and difficulties in scheduling appointments with work. These factors weighed heavily on the decision to continue or discontinue PrEP use. These results are consistent with previous studies that identified that abandonment or irregular use of prophylaxis is strongly linked to the accumulation of logistical challenges, such as long commutes, long waits, and lost workdays, even when the medication is free [52, 54–56]. Therefore, the location of services and the frequency of visits directly affect adherence. These findings suggest the importance of decentralizing and facilitating access to primary care, promoting flexible hours, expanded geographic coverage, and a continuous supply of medication, because barriers such as waiting times and travel, although commonplace, have a real impact on the decision to continue or discontinue prophylaxis. The positive perceptions of PHC observed in this study should be seen as the result of specific contexts directly associated with the performance of well-prepared teams, respectful communication, and the guarantee of privacy, rather than as characteristics inherent to all primary care services. Among the study’s original aspects, participants’ preference for PHC services over specialized services is notable, with the former associated with greater discretion and reduced exposure to stigma. Stigma remains a considerable barrier to engagement in HIV prevention, both globally and in Brazil, being associated with the fear of being labeled, judged, or simply recognized as belonging to certain groups [57–60]. Welcoming, judgment-free care relationships emerged as important elements important elements for ensuring continued use, which is directly related to research correlating respectful counseling with PrEP adherence [61–65]. In contexts where the FHU is perceived as a space for expanded care, not restricted to HIV, PrEP use tends to normalize, reducing the potential social risks associated with public exposure. However, this potential depends on trained teams, privacy safeguards, and institutional competence. Without these, PH can, paradoxically, reinforce stigma.

Stigma and moral judgments, operating at the interface between popular and moral sectors, remained present throughout the participants’ TI, associating PrEP use with promiscuity or hidden HIV infection, leading many to conceal their use and restrict the dissemination of information about the medication. This phenomenon, documented in the literature, demonstrates that conservative social norms, mistaken associations between PrEP and HIV, and fear of judgment compromise adherence and re-engagement [66, 57, 59]. Conversely, trust, fostered by safe environments of dialogue, ongoing counseling, and peer support, encourages continued use and open communication about PrEP [61, 67]. Thus, strengthening the relational dimensions of care, including welcoming practices and ensuring confidentiality, is as crucial as ensuring medication availability.

Another recurring aspect in the reports was the occurrence of pauses and resumptions in PrEP use, generally linked to changes in relationships, professional routines, or personal perception of risk, but without indicating definitive abandonment. This pattern aligns with the idea of “effective adherence,” proposed by recent international debates, according to which PrEP is used intermittently, during periods of greater exposure to HIV [68–72]. Examining the TI makes these transitions visible, suggesting that PrEP programs should support safe interruptions and resumptions, provide clear guidance, enable agile return flows, and adopt service models designed to eliminate excessive bureaucracy. Intermittent use of PrEP challenges traditional conceptions of adherence based on rigid and uninterrupted treatment continuity, pointing to the need for a more situated and contextualized understanding of health engagement. Furthermore, understanding intermittency as a rational strategy demands flexible responses. It would be interesting if this perspective implied a reorientation of health practices and policies, so as to embrace these transitions as a legitimate part of care [73, 74].

Some participants used reminders to avoid forgetting their medication or negotiated condom use flexibly, showing that PrEP integrates with broader and more contextual prevention strategies. Studies indicate that it works in a complementary way to sexual protection practices and STI care, and that counseling interventions should combine prevention, realistic discussions about condoms, and non-judgmental listening, recognizing that sexual choices change according to context [75–77].

Some limitations of the study need to be highlighted. Because this is a qualitative study based on in-depth interviews, it is important to recognize that the collected accounts reflect personal experiences and are therefore subject to memory and social desirability biases, despite the methodological efforts undertaken to mitigate these effects. The data are concentrated in a single FHU in Manaus, bringing together a restricted group of participants, mostly cisgender men already using PrEP. This limits the generalizability of the findings and may exclude profiles that face greater barriers to access. Thus, the obstacles identified may even underestimate the structural and social difficulties faced by those who never managed to start PrEP or discontinued it before even the first dose. Added to this is the fact that aspects such as socioeconomic conditions, transportation options, and the organization of health services themselves were not systematically controlled, variables that can directly influence the narrated experiences. In the context, elements associated with the folk sector emerged only sporadically, reinforcing the emphasis on institutional dynamics. It becomes evident, therefore, that future research in varied contexts, including people at different stages of contact with PrEP, is fundamental to supporting more comprehensive policies capable of addressing unmet needs and promoting greater equity in HIV prevention.

Kleinman’s theoretical framework could be expanded to consider multiple actors involved in the production of care; however, this study focused exclusively on PrEP users’ perspectives to understand how they experience and negotiate their TI during prophylaxis. Thus, the perceptions of healthcare professionals, family members, peer networks, and other actors linked to the professional and popular sectors were not directly explored. The study’s intention was not to reconstruct the full dynamics of the various sectors of care, but rather to analyze how these sectors are perceived, experienced, and traversed by PrEP users throughout their TI. Future research that includes multiple actors may contribute to a broader understanding of these itineraries and the interactions between the different sectors of care.

Furthermore, although the sample included a transgender man, there was no participation of transgender women in the interviews, despite this population being disproportionately affected by HIV. This absence limits the diversity of perspectives presented and restricts a more comprehensive understanding of TI in groups facing more significant historical barriers to HIV prevention and care.

Despite these limitations, the results offer valuable insights into how PrEP is experienced and negotiated within PHC in the Amazon region. The study enriches discussions on the decentralization of HIV prevention, continuity of care, and the social dimensions of PrEP use, highlighting how TI are constructed from everyday experiences, social relationships, institutional practices, and access conditions.

## Conclusion

By highlighting the TI and adopting Kleinman’s Health System model, this study shows how personal experiences, social bonds, moral values, and the very organization of health services intertwine and shape the use of PrEP over time. The findings primarily illuminate the popular and professional dimensions of care, while elements related to the folk sector emerged less consistently in participants’ narratives. For PHC to truly fulfill a strategic role in HIV prevention, it is not enough to simply expand access; it is necessary to confront structural barriers, combat stigma, guarantee confidentiality, and value the relational dimensions of care. This may support the broader implementation of PrEP as a truly equitable, flexible, and sustainable public strategy in the Amazon and similar realities.

More than an empirical contribution, this study offers valuable insights for those who design public health policies and organize services dedicated to HIV prevention. By closely examining the TI of people using PrEP in primary care, it became evident that decentralizing this care not only reduces stigma but also facilitates ongoing relationships and creates space for more integrated approaches that are truly centered on everyone’s needs. The evidence presented here points to practical possibilities for managing the SUS, such as simplifying clinical and administrative procedures, eliminating persistent bureaucratic obstacles, and investing in confidentiality as part of routine care. Although this work is set in the Brazilian Amazon, the lessons learned strongly resonate with other contexts marked by social vulnerability or a scarcity of specialized services, reiterating the role of primary care as a strategic foundation for expanding access to PrEP in universal health systems.

## Supplementary Information

Supplementary Material 1

## Data Availability

All data supporting the findings of this study are available within the paper and its Supplementary Information.

## Abbreviations

AIDS: Acquired immunodeficiency syndrome
CAAE: Certificado de Apresentação de Apreciação Ética
COREQ: Consolidated Criteria for Reporting Qualitative Research
FHU: Family Health Unit
FMT: HVD–Fundação de Medicina Tropical Doutor Heitor Vieira Dourado
HIV: Human Immunodeficiency Virus
IDI: In–depth Interviews
MSM: Men who have Sex with Men
PEP: Post–Exposure Prophylaxis
PHC: Primary Health Care
PrEP: Pre–Exposure Prophylaxis
RFS: FHU–Dr. Raimundo Franco de Sá Family Health Unit
SSIG: Semi–Structured Interview Guide
STI: Sexually Transmitted Infection
SUS: Sistema Único de Saúde (Brazilian Unified Health System)
TI: Therapeutic Itineraries
FHU / FHUF: Unidade Básica de Saúde / Unidade Básica de Saúde da Família
FHU: Unidade de Saúde da Família
WHO: World Health Organization
