# Supplementary material for: Pre-exposure prophylaxis (PrEP) in primary health care: A qualitative study of users’ therapeutic itineraries in the Brazilian Amazon
Source: BMC Public Health. 2026 Jul 22;26:2994. doi: 10.1186/s12889-026-28273-5 (PMC13679125; doi:10.1186/s12889-026-28273-5)
Supplement: Supplementary file 1 [file 12889_2026_28273_MOESM1_ESM.docx]

**SUPPLEMENTARY MATERIAL 1**

**IN-DEPTH INTERVIEW GUIDE/QUESTIONNAIRE:**

**Search for pre-exposure prophylaxis (PrEP) for HIV/AIDS in the Brazilian Amazon: a qualitative study of patients' therapeutic journey**

| SEMI-STRUCTURED QUESTIONNAIRE | OBJECTIVES |
| --- | --- |
| 1. How did you learn about PrEP? | (General perceptions) Describe the level of knowledge users have about PrEP, their motivations, and the factors that influence their decision. |
| 2. What explanation or information did you receive that led you to decide to use PrEP? |  |
| 3. Are you currently using it, or have you ever used it? Where? For how long? |  |
| 4. What is your opinion about PrEP? What do you think about its reliability? |  |
| 5. What factors or reasons influenced your decision to use PrEP? |  |
| 6. Could you talk about how PrEP adds value, or has added value, to you and your sexual practices? | (Perceived value/impact) Describe the perceived impact of PrEP use and unsafe sexual behavior.  (What is the impact on users' lives, changes in behavior, whether or not they adopt, for example, greater adherence to testing, combination of other tools as advocated by 'combined prevention', or whether they start to have behaviors that may put them at greater risk than before PrEP). |
| 7. What do you think of the exams and tests that accompany the PrEP routine? |  |
| 8. Describe how you consider the importance of follow-up and return to the PrEP service? Did the doctor or nurse discuss this with you? |  |
| 9. For you, is PrEP associated with any prejudice and/or stigma in any way, according to your experience? Could you talk a little about it? And do you think this can influence adherence in any way? |  |
| 10. Could you say what it's like or how you feel about exercising your sexuality and sexual practices while using or after using PrEP? |  |
| 11. What do you think would lead a person to abandon PrEP? |  |
| 12. Have you ever forgotten to take your medication? What do you do to avoid forgetting to take your medication? |  |
| 13. What do you think about the possibility of injectable PrEP? If it were offered to you, would you accept it depending on the frequency, such as with contraception for women (once a month)? |  |

| 14. How is your experience using PrEP? How do you feel while using it? (describe possible advantages and disadvantages during use) | (Emotional perception) Describe feelings related to medication use, for example: fear, discrimination, safety, insecurity, etc. |
| --- | --- |
| 15. Within your social circle, family and partners, how are you viewed when using PrEP? What kind of comments do you hear? |  |
| 16. How do you think people generally view PrEP users? (describe if there is prejudice, discrimination, stigmatization, association with HIV infection) |  |
| 17. Would you say that stigma and prejudice about HIV/AIDS can be barriers to PrEP use? And do you consider that this can influence adherence in any way? |  |
| 18. How do you view the dissemination of this prophylaxis to the public? (How should it be?) | (Access, itinerary and infrastructure)  Describe access to PrEP, itineraries, the flow of available information, dissemination and means of communication, infrastructure offered, staff, access to medication, and understand if it affects professional, personal and social routines. Understand if there is any difference between receiving PrEP in a specialized unit and a basic health unit. |
| What is the route from your residence to the primary health care unit? How long does this journey take you? What means of transport do you use to get to the primary health care unit? |  |
| 20. Do you need to miss work, school, or university to attend your PrEP appointment? Has there ever been a situation at work, school, or university that prevented you from attending your PrEP appointment? |  |
| 21. What is the situation at your company, school, or university regarding those who have been tested? |  |
| 22. Can you describe what access to PrEP is like? Are there any difficulties? And what are the biggest difficulties in finding and using PrEP for you? |  |
| 23. Do you think there is any difference between receiving PrEP in a specialized unit and in a basic health unit? |  |
| 24. How would you describe the infrastructure of the place and the care provided by the team of professionals where you receive PrEP? |  |
| 25. Do you have any suggestions for improving the PrEP service in your city? |  |

**SUPPLEMENTARY MATERIAL 2**

**Consolidated Criteria for Reporting Qualitative Studies (COREQ): 32-item checklist**

Developed from:

Tong A, Sainsbury P, Craig J. Consolidated Criteria for Reporting Qualitative Research (COREQ): A 32-Item Checklist for Interviews and Focus Groups. *International Journal for Quality in Health Care* . 2007. Volume 19, Number 6: pp. 349–357

| **Item number** | **Questions/Guide Description** | **Reported on page #** |
| --- | --- | --- |
| **Domain 1: Research team and reflexivity** |  |  |
| *Personal characteristics* |  |  |
| 1. Interviewer/facilitator | *Which author(s) conducted the interview or focus group?*  PSA | Page 8. |
| 2. Credentials | *What were the researchers' credentials?*  Doctorate, Medicine , Bachelor's Degree, Master's Degree, Master's in Research | Page 8. |
| 3. Profession | *What was their occupation at the time of the study?*  Doctors, master's students, doctoral students, and qualitative researchers. | Page 8. |
| 4. Gender | *Was the researcher male or female?*  Some researchers were men, others were women. | Page 8. |
| 5. Experience and training | *What experience or training did the researcher have?*  Renowned researchers, as well as graduate students with experience in qualitative research, all with training in the field and published articles. | Pages 8-9. |
| *Relationship with participants* |  |  |
| 6. Established relationship | *Was there any established relationship before the start of the studies?*  None of the participants had a prior relationship with an author before the start of the study. | Page 9. |
| 7. Participants' knowledge about interviewer | *What did the participants know about the researcher? (e.g., personal goals, reasons for conducting the study) to look for ).*  The participants did not know the interviewer beforehand. They were invited to participate in the study while receiving care at the RFS primary health care unit. | Page 6. |
| 8. Interviewer Characteristics | *What characteristics were reported about the interviewer/facilitator? For example, bias , assumptions, Reasons and interests in the research topic .*  No interviewer-related biases were identified. | Page 8. |
| **Domain 2: Study design** |  |  |
| *Theoretical framework* |  |  |
| 9. Methodological guidance and Theory | *What was the stated methodological approach used to support the study? For example, grounded theory, Discourse analysis, ethnography, phenomenology, content analysis .*  The research team members ( PA, PB, DB ) independently conducted a thematic analysis using a predominantly inductive approach. | Page 8. |
| *Selection of participants* |  |  |
| 10. Sampling | *How were the participants selected? Ex.: purposeful, convenient, consecutive, snowball effect .*  Intentional | Page 7. |
| 11. Approach Method | *How were the participants contacted? For example, in person, by phone, by mail, or by email .*  The first contact with all participants was made by telephone, following an in-person explanation of the research. | Page 7. |
| 12. Sample size | *How many participants were in the study?*  20 PrEP users | Page 9. |
| 13. Non-participation | *How many people refused to participate or dropped out? What were their reasons?*  No one has given up on participating in the project. | N/A |
| *Context* |  |  |
| 14. Data collection configuration | *Where was the data collected? For example, at home, at the clinic, at the workplace .*  The interviews were conducted by PA in a private, secure, and quiet room to maintain anonymity and avoid excessive noise and discomfort. | Page 8. |
| 15. Presence of non-participants | *Was there anyone else present besides the participants and researchers?*  No, only the interviewer was in the room during the interview. | Page 8. |
| 16. Sample description | *What are the important characteristics of the sample? for example, demographic data, date*  These data are described in the study results. | Page 9. |
| *Data collection* |  |  |
| 17. Interview Guide | *Did the authors provide questions, suggestions, and guidance? Was the project tested in a pilot study?*  The questions were developed by the Research Team and previously tested and validated by the researchers on a smaller sample of volunteers, with adjustments made to ensure the transparency and relevance of the questions. | Page 8. |
| 18. Repeat interviews | *Were repeat interviews conducted? If so, how many?*  No | N/A. |
| 19. Audiovisual recording | *Did the research use audio or video recordings to collect the data?*  Audio recordings. | Page 8. |
| 20. Field notes | *Were field notes taken during and/or after the interview or focus group?*  No | N/A. |
| 21. Duration | *How long were the interviews or focus group?*  The interviews lasted an average of 45 minutes. | Page 8. |
| 22. Data saturation | *Was data saturation discussed?*  The number of interviews was determined by the principle of theoretical saturation, in which interviews are conducted until a clear pattern emerges and subsequent groups do not produce new information. | Page 8. |
| 23. Returned transcripts | *Were the transcripts returned to the participants for comments and/or corrections?*  No. | N/A. |
| **Domain 3: Analysis and conclusions** |  |  |
| *Data analysis* |  |  |
| 24. Number of data encoders | *How many data encoders encoded the data?*  Three researchers independently developed a codebook and performed the coding line by line. | Page 8. |
| 25. Description of the coding tree | *Did the authors provide a description of the coding tree?*  No. | N/A |
| 26. Derivation of themes | *The themes were identified in advance or* *derived from the data?*  The analysis of the interviews allowed us to identify three main themes. | Page 9. |
| 27. Software | *What software, if applicable, was used to manage the data?*  MAXQDA 20 Program | Page 8. |
| 28. Participant Verification | *Did the participants provide feedback on the findings?*  No. | N/A |
| *Reports* |  |  |
| 29. Quotes presented | *Were quotes from the participants presented to illustrate the themes/discoveries? Each one was... Citation identified? Ex.: participant number*  Yes, quotations were presented to illustrate the themes/findings, and each quotation was identified with a participant number. | Pages 14-34. |
| 30. Consistent data and conclusions | *Was there consistency between the data presented and the conclusions?*  Yes, there was consistency between the data presented and the conclusions. | Pages 14-38. |
| 31. Clarity of main themes | *Were the main themes clearly presented in the results?*  Yes, the main themes were clearly presented in the Results section, using specific sections for each theme. | Pages 14-34. |
| 32. Clarity of secondary themes | *Are there any descriptions of various cases or discussions of secondary topics?*  Yes, secondary topics were discussed. | Pages 14-38. |

**SUPPLEMENTARY MATERIAL 3**

**List of Codes**

| **1. Entry points and knowledge about PrEP** |  |
| --- | --- |
| **1.1 Introduction to PrEP** | **37** |
| **1.2 Dissemination strategies** | **18** |
| **1.3 Knowledge about HIV/AIDS** | **23** |
| **1.4 Understanding PrEP** | **75** |
| **1.5 Social importance of PrEP** | **45** |
| **1.6 Doubts and uncertainties** | **23** |
| **1.7 Knowledge about other STIs** | **19** |
| **1.8 STI Screening** | **26** |
| **1.9 PEP as an entry point** | **8** |
| **1.10 Incentives to start PrEP** | **66** |
| **2. Navigation and start of service** |  |
| **2.1 Access to the PrEP service** | **41** |
| **2.2 Ease of access to services** | **57** |
| **2.3 Dispensing and access to medication** | **23** |
| **2.4 Humanized service** | **15** |
| **2.5 Skilled professionals** | **9** |
| **2.6 Unprepared professionals** | **13** |
| **2.7 Refusal or institutional barriers to care** | **61** |
| **2.8 Stigma and prejudice in health services** | **5** |
| **2.9 Social network support** | **19** |
| **3. Monitoring and transitions in care** |  |
| **3.1 Clinical monitoring of PrEP** | **106** |
| **3.2 Regularity and adherence to PrEP use** | **72** |
| **3.3 Duration of PrEP use** | **22** |
| **3.4 Changes in habits and behavior** | **58** |
| **3.5 Impact on mental health** | **44** |
| **3.6 Safety and confidence in PrEP** | **84** |
| **3.7 Adverse effects** | **37** |
| **3.8 Alternative prevention methods** | **40** |
| **3.9 Sexual behavior** | **54** |
| **3.10 Injectable PrEP** | **40** |
| **4. Points of friction in the therapeutic itinerary** |  |
| **4.1 Geographic or structural access barriers** | **61** |
| **4.2 Organizational barriers to service** | **94** |
| **4.3 Lack of information about PrEP** | **39** |
| **4.4 Stigma and social prejudice** | **105** |
| **4.5 Lack of social support** | **66** |
| **4.6 General difficulties in using or continuing PrEP** | **39** |
| **5. Discontinuation and resumption of care** |  |
| **5.1 Discontinuation of PrEP use** | **59** |
| **5.2 Reasons for interruption** | **38** |
| **5.3 Resumption of PrEP use** | **19** |
| **5.4 Factors that promote continuity of care** | **33** |

**Supplementary Material 4**

**Full interview excerpts organized according to Kleinman’s Health Care System model**

This supplementary material presents all interview excerpts in full, organized according to the analytical categories derived from Kleinman’s Health Care System model (popular, professional, and folk sectors), as well as cross-sectoral themes.

**1. Popular Sector**

The popular sector encompasses everyday knowledge, meanings, motivations, and social influences that shape the initiation and continued use of PrEP. Family relationships, friendships, media exposure, and personal experiences play a central role in how individuals interpret HIV risk and prevention strategies.

**1.1 Initial knowledge about PrEP**

*“The first time I heard it was on the news. I was watching a report, that’s when it appeared”* (Participant 10 - cis woman - heterosexual - serodifferent partnership)

*“Well, it was through Instagram. I was interested; I didn’t know it existed until then. I spoke to a colleague in the health field. He recommended a FHU to me (...), and I went straight there. I had my first consultation, got the medication, and started taking it”* (Participant 2 – trans man - bisexual)

*“I heard about it through the campaign of a political candidate who was doing some promoting it* *, so I did some more research, and it was he who gave me the recommendation for the health unit”* (Participant 13 - cis man - homosexual)

*“Well, I found out about PrEP through a friend. He is a nurse and was already using it, (...) I asked him. I started asking around, (...) People say: 'PrEP is this, that'. I was curious, and I tried it”* (Participant 9 - cis man - homosexual)

*“Through a friend. He used PrEP and it helped prevent HIV (...) He had a serodifferent partner. I researched the subject. I trusted him and I still use it today”* (Participant 15 - cis-bisexual man)

*“I have two friends who were taking it and then I became interested. I went to find out how it worked. (...). I went to the place to start. I talked to the girl (nurse), and she let me take it due to the circumstances”* (Participant 18 - cis woman - bisexual)

*“PrEP, my husband is positive. So, I went with him to the appointment, and that's when the lady (nurse) said that there was a follow-up for people who were not positive, if I agreed to participate”* (Participant 11 – cis woman - heterosexual - serodifferent partner)

*I learned about it through a situation I experienced with my son’s father (...) I discovered that he had cheated on me. Through this cheating, he contracted HIV. And when he confessed to me, I was in despair. (...) but I went to take the test, and mine was negative. That’s when the girl (nurse) introduced me to PrEP”* (Participant 06 – cis woman - heterosexual - serodifferent partnership)

**1.2 Personal motivators for starting and adhering to PrEP**

*“I had sex with a partner and had sex without a condom. So, I ran to the hospital to use PEP. The doctor told me that after I finished the PEP treatment, I should look for PrEP”* (Participant 8 - cis man - homosexual)

*“I think it’s good that I get (PrEP). It’s good for my health. Yeah, I don’t have to worry”* (Participant 20 - cis woman - heterosexual - serodifferent partner)

*“I had met someone. We were getting along well, and he decided to open up to me. He said he had been living with HIV since 2014. That was the icing on the cake for me to decide to start PrEP”* (Participant 14 - cis man - homosexual)

*"When I met my current husband, he wanted to stop his HIV treatment, and I forced him to do it. He took me to the basic unit where he was receiving treatment, I had to take the tests, and the woman said I could use PrEP (...)* (Participant 10 - cis woman - heterosexual - serodifferent partner)

*“At first. When I found out (She found out her husband had HIV) ... My world fell apart. But today, taking the test, pricking my finger, is normal (...) For me, I have no difficulty. I trust the medication. And it works*” (Participant 19 - cis woman - heterosexual - serodifferent partner).

*“I accepted it out of desperation, out of fear of contracting HIV. Fear of taking a test and finding out that I had contracted the virus. As soon as the nurse told me to, I immediately accepted it. I didn’t think twice. I just wanted to get rid of it. I just wanted to prevent it. I just wanted to have the minimum risk of contracting it”* (Participant 6 – cis woman - heterosexual - serodifferent partner)

*“(...) I’m very intense. I have a very active sex life (...) I was afraid, very afraid. I did things and felt guilty”* (Participant 15 – cis man - bisexual)

*“I was getting involved with a lot of people (...) I’m very nervous about illnesses, about anything, but we know that at some point, it could happen. (...) I kept thinking: ‘Oh my God, what if this happens to me (...)’ and I was getting involved with people I didn’t know very well or who were just flings. I decided to take it because at least I will have more security (...)”* (Participant 18 – cis-bisexual woman)

*“I’m bisexual. I have relationships with anyone I’m attracted to. So, because I have a very active sexual life, I looked for PrEP”* (Participant 2 – trans man - bisexual)

*“I was getting involved with people I didn’t know very well, or who were just flings and flings. I said, oh, I’ll take it because at least I’ll have more security, right?”* (Participant 18 – cis woman - bisexual)

*“(…) I trust it a lot. Because I’ve already had three experiences with three people I was in a relationship with, and they got infected with HIV and I didn’t.”* (Participant 12 – cis-homosexual man)

**1.3 Understanding of prophylaxis**

*“It is a pre-exposure medication, if you are exposed to the virus, there is	less chance of catching it”* (Participant 18 – cis-bisexual woman)*.*

*“It protects against HIV (...), it is 99% effective and cannot fail. It is used daily”* (Participant 06 – cis woman - heterosexual).

*“My husband is positive. I went with him to the appointment, and the nurse said that there was treatment for people who were not positive. (...) I had never heard of this treatment”* (Participant 11 – cis woman - heterosexual - serodifferent partner).

*“Well, I didn’t know about PrEP. I’ve been with my husband for 18 years. There was an affair, and he found out he had HIV (...) My result was negative when I took the rapid test to determine if I was infected. The nurse told me about PrEP”* (Participant 19 – cis woman - heterosexual - serodifferent partner).

*“I am fully aware that PrEP protects against HIV, it does not protect against other diseases”* (Participant 13 – cis-bisexual man).

*“PrEP (...) must be used with a condom. Because condoms break. And if there is a problem, the person will be infected with HIV. But condoms can also be used for other types of diseases. Hepatitis, syphilis, and hepatitis. Condoms work for any disease. But PrEP is specific for HIV”* (Participant 12 – Cis Man – Homosexual).

*“I use condoms, but it gave me more freedom (...) I relaxed the rules in this case”* (Participant 03 – cis man - homosexual)

*“I'm allergic to latex... So, for me, using condoms is complicated. So, I preferred to protect myself against HIV this way, but (...) STIs are complicated. HIV is the worst. So, I preferred to protect myself mainly from HIV. And the others... I'm afraid”* (Participant 12 – cis man - homosexual)

*“After I started using (PrEP), I went overboard with condoms. I hardly use them. And then I think about it. I don't know the person. But I took the risk”* (Participant 15 – cis man - homosexual)

*“I stopped for a few periods in these six months, but I always go back. I got into a monogamous relationship (…) I felt comfortable and trusted the person”* (Participant 3 – cis man - homosexual)

*“PrEP will be with me, regardless of whether or not I have a partner, it is an extra shield for me, for my good, so it is something I will continue”* (Participant 4 – cis woman - heterosexual - serodifferent partner)

*“I already asked my doctor. She said there was a fee, well, but of 99 reais and something. Quite high, in fact. I have this confidence that it works”* (Participant 2 - trans man - bisexual)

*"Well, we have to trust, don't we? Every month when it's time to go for the exam, I get anxious (...) Whether I like it or not, I don't know”* (Participant 11 - cis woman – heterosexual)

**2. Professional Sector**

The professional sector encompasses formal health services, including infrastructure, organization of care, health professionals, and follow-up routines. This sector highlights how access, service design, and quality of care influence adherence and continuity of PrEP use.

**2.1 Access and resources needed**

*“I spoke to a colleague who works in the health field, and he recommended a FHUF to me, which was Franco de Sá. I went straight there”* (Participant 2 - trans man - bisexual)

*“It’s far from my house. I go by public transportation. It takes about 25 minutes to get there. When there aren’t many people, I can get everything done in 1 to 1.5 hours and then return home. There’s a PrEP room where I also wait until I’m seen, and there are many people. It takes a while, but it’s worth it”* (Participant 15 – Cis man – bisexual)

*“I go by car... There are places that are closer to my location, but they don’t have PrEP”* (Participant 18 – cis woman – bisexual)

*“I walk (...) from my house to the FHU, it takes about half an hour (...) This whole walking thing is really hard (...) I think that for me, the closer the better”* (Participant 10 – Cis woman – heterosexual - serodifferent partner)

*“If there was a FHU near my house... Oh my God, that would be great! Because it’s very close. I went there to ask, but they didn’t even know what PrEP was. Because if it were there, I would be there.”* (Participant 08 – Cis man – Homosexual)

*“I go out during my lunch break (...) It’s far. I don’t know how to take the bus. Since I don’t know how and the route is on the wrong side of the road, I always take Uber. The bus costs R$4.50. If we go by bus, we must pay two or three fares. So, it becomes more expensive”* (Participant 11 – Cis woman – Heterosexual - HIV-discordant partner)

*“I’m currently unemployed; there are days when I don’t even have money to eat, let alone pay for the bus fare to get PrEP”* (Participant 10 – Cis woman – Heterosexual - HIV-discordant partner)

**2.2 Health care and service preferences**

*“Here in the* Referral unit *for HIV treatment, it was harder to adhere, it’s more bureaucratic. The doctor said it would be more accessible at the FHU. That’s when I met another guy using PrEP, who recommended Franco de Sá to me (...). We did the rapid tests when I got there, and everything was fine. Then they give you a vial for 30 days”* (Participant 08 – Cis man – Homosexual)

*“I came here (Tropical Medicine Hospital, Referral unit for HIV treatment), and I found it more difficult because I had to make an appointment with the infectious disease specialist. It was scheduled, but I couldn’t make it that day. There (FHU), I arrived, went, and told the nurse that I wanted to take PrEP, and I was seen right away and got the medication the same day”* (Participant 0 – Cis man – Homosexual)

*“I called here (Tropical Medicine Hospital, Referral unit for HIV treatment), but they told me it was too bureaucratic, that's when they recommended the FHU, even though it's a bit far away, but when I got there for the first time and registered it was straightforward. So, I think having the FHU makes the issue of distance more accessible because not everyone lives near this region”* (Participant 13 – Cis man – Homosexual)

*“There is a professional there who is very good. He takes good care of us. We feel comfortable talking about things. He is not in a bad mood. As it happened, I arrived at a health center with secretions from my penis and the professional was super rude to me.”* (Participant 07 - cis man - homosexual)

*“I take the exams every four months. (...) This last time I went, I felt even more welcomed. There is a nurse who gives you all the attention”* (Participant 11 – cis woman - heterosexual - serodifferent partnership)

**2.3 Service and follow-up time**

*“I get a ride with my father on the way there. From there, I usually go back to work. The most I’ve ever spent at the FHU was an hour. But because there are people in front of me, the screening itself, the exam, everything is rapid. Yes, even to get the medicine” (Participant 2 – Trans man – bisexual)*

*“At one o’clock in the afternoon, you go there. The appointments are on a first-come, first-served basis. You don’t need to get a form. There’s a specific office for that. So, you get there, and people sit outside the FHU, like in a hallway. Then at one o’clock in the afternoon, they start calling you, do a rapid test, talk to you, and enter your information. You go to the other nurse who writes the prescription. You go to the pharmacy, pick it up, that’s it, and you leave.”* (Participant 16 – Cis man – homosexual – serodifferent partnership).

*“It’s just all this work. It’s bureaucracy. But I also understand that it’s part of the SUS, which is very high demand. But it would be better if we left the office with the exam already scheduled, to collect the sample”* (Participant 7 – Cis Man – Homosexual)

*“We do the quick tests. We go to the doctor, to the nurse. He asks a lot of questions… And he gives us the medicine, but we must go back to do the tests. Which is the complete blood count, HIV, syphilis, hepatitis, kidney function… I think it's great!”* (Participant 8 – cis man – homosexual)

*“I think it’s great, because before taking PrEP, I would have annual exams. … sometimes for a year and a half without doing anything (...) if there’s something you’ll only know if it’s very advanced. And if I go there every 4 months, I’ll know and take care of it”* (Participant 18 – cis-bisexual woman)

**2.4 Consultation, medication dispensing, and daily use**

*“I usually go in the morning for a consultation or exam. Then I go to work. (…) When they schedule an afternoon appointment (consultation or exam), I ask to leave work. I go and come back (…) it’s easy”* (Participant 02 – trans man - bisexual)

*“I go out for lunch and take an Uber from the front of my workplace to the Health Unit. I have 2 hours to go and come back, and when I go over the appointment time, I leave work later.”* (Participant 11 – cis woman – heterosexual – serodifferent partner)

*“The work issue bothers me. They only give me a certificate of attendance for those hours I am at the appointment, and I must return to work later. Sometimes, I can't go back... For example: If I go in the afternoon... To schedule the exam... Which must be done in the afternoon... I can't go back in the afternoon anymore. And if I must do it in the morning... I have to go back right away”* (Participant 8 – Cis man – homosexual)

*"I think the infrastructure of the health unit is good; sometimes it gets quite crowded in the morning, but this is common in all FHUs"* (Participant 1 – cis man – homosexual)

*"I think the infrastructure is cool. There are laboratories there, there are professionals available, there are computers, a pharmacy. I think it's good" (Participant 3 – cis man – homosexual)*

*"The nursing technician who helps me do the rapid test is a very nice person (...) The nurse is also a wonderful professional"* (Participant 04 - cis woman - heterosexual - living in a serodifferent partnership)

*“Many people watch the FHU pharmacy where we get our medication. They hand us everything. The pharmacy was more reserved at the other unit we used to go to. We would go in, give them the paper, and they would give us the PrEP in a box. There weren’t many people looking”* (Participant 05 – cis woman – heterosexual – serodifferent partner)

*“I take it every night. I prefer to take it at night (...) at eight-thirty o`clock. If I go out somewhere, I put a pill in a tiny container, and it goes with me in my bag. When it’s eight thirty, my cell phone wakes up wherever I am. It’s time to take my medicine.”* (Participant 4 – Cis woman – Heterosexual - HIV-discordant partner)

*“Sometimes I forget. It’s normal for me to forget one day. Especially since I take it at night. I usually take it at 11 p.m. That’s when I’m already home. I take it and go to sleep. But sometimes, I drink alcohol and avoid taking it. I prefer to avoid it. I take it the next day. As soon as I wake up, I take it. Then, at night, I retake it so I can continue the cycle.”* (Participant 12 – Cis man – homosexual)

*“I try to take it every day. Between 9 pm and 10 pm. I’m starting to write it down every time I take it. I go there and write it down. I forget a lot. I started writing it down now because I was always in doubt”* (Participant 8 – Cis man – homosexual)

*“I bought a little box with dividers for the days of the week. On Mondays I go there and take it, on Tuesdays I go there and take it and then on Sunday when it’s all gone, I refill it. This makes things much easier. It was my strategy not to miss doses and maintain care. That’s why I also said that PrEP has become a form of self-care”* (Participant 14 – Cis woman – Heterosexual - HIV-discordant partner)

**2.5 Criticism and suggestions**

*“When you get the bottles, you must carry them in your hand... Or you put them in your pocket... Or if you have a bag... But if we don't carry a bag... there are several rather large bottles. If there was a small bag that wasn't transparent, it would be more comfortable for some. Because people stare”* (Participant 08 – cis man - homosexual)

*“We have this difficulty. Because we need to go and schedule an appointment. To schedule the exam, go on the day of the exam, take the exam, and schedule a new one. Every 4 months I must go there 3 times. If it were just one day, it would be good” (Participant 3 – Cis man – Homosexual)*

*“My only criticism is regarding the delay in starting the service. Because when we get there, the door is always closed. There is no one to inform us of anything, we don’t know if there are people inside. We only know when someone decides to open the door and starts calling. (…) I think it’s a lack of communication with the patients” (Participant 2 – Trans man – bisexual)*

**3. Folk Sector**

The folk sector refers to informal norms, stigma, moral regulation, and social beliefs surrounding HIV prevention and PrEP use.

**3.1 Prejudice and discrimination**

*“I realize there is prejudice because people immediately think, ‘Look, this girl is for everyone!’. I think people see promiscuity. I think they see people as sluts”* (Participant 18 – cis woman – bisexual)

*“I told a colleague. He said: it’s not for you to be doing this, because it’s only for people like this, like that, like that (pejorative sense). Or are you like that?”* (Participant 15 – cis man – bisexual)

*“They either think I’m a prostitute or that I have risky sexual exposure. That I’m promiscuous, a slut all the time, or that I live with someone who has HIV”* (Participant 14 – cis man – homosexual)

*“I ended up talking to some people about PrEP. The first question was: but why you? Aren’t you married? Don’t you live with your husband? So, I don’t want to expose him, you know”* (Participant 14 – cis man - homosexual)

*“I realize there is prejudice because people immediately think, ‘Look, this girl is for everyone!’. I think people see promiscuity. I think they see people as sluts”* (Participant 18 – cis woman – bisexual)

*“I told a colleague. He said: it’s not for you to be doing this, because it’s only for people like this, like that, like that (pejorative sense). Or are you like that?”* (Participant 15 – cis man – bisexual)

**3.2 Communication and trust**

*“(...) I'm very open; I told my parents about PrEP. They were shocked, but it was okay, I'm taking care of myself (...). Usually, when it's a friend, as soon as I trust them, (...) who needs it, or who I have a good relationship with, I tell them”* (Participant 18 – cis-bisexual woman)

*“People get very curious when I talk (...) I talk excitedly (...) I talk about how it works, and I've already influenced a lot of people who I think need to use PrEP”* (Participant 9 – cis-homosexual man)

**4. Across sectors: Facilitators, barriers, and patterns of PrEP use**

**4.1 Facilitators**

*“I feel safer, mainly because I’m not in a relationship with just one person. (Participant 1 – cis homosexual man)*

*“After we start taking it, we feel more available , but at the same time, we are a bit wary, so we protect ourselves more. (Participant 18 – cis bisexual woman)*

*“It helps us take care of ourselves (...) I didn’t take care of myself (...) after we start taking the medication, we feel freer” (Participant 15 – cis bisexual man)*

*“The fact that I go to the FHU every three months as if it were my obligation makes me feel more confident about taking care of other things too. So, I started taking better care of myself, so I asked the nurse: I want to see the doctor, you know? Things that I didn't usually do. So, I take advantage of it and do it”* (Participant 15 – cis-bisexual man)

*“Seeing the doctor also means taking better care of my health. Because whether you like it or not, it's something like that that encourages you to pay a little more attention to it. And then when you start going to everything there, you start to understand that it's important for me to pay attention”* (Participant 13 – cis-bisexual man)

*“I stopped for a while (stopped using PrEP). I took a break. As a salesperson, I’m pressured to produce more (...) There came a point where the company was apprehensive, with a lot of people being fired. I didn’t want to leave my job to seek treatment because I was afraid of being frowned upon” (Participant 13 – Cis man – homosexual)*

**4.2 Barriers**

*“There is a lot of prejudice. My son is going to be 17, he might want to stay away from us (because they are in a serodifferent relationship and one of them uses PrEP). We never talk to other people about this. When I go to the doctor, I am separated from my husband. Sometimes, I go by and hear: this woman is going to take PrEP. Or this man is going to take this HIV medication.”* (Participant 5 – cis woman - heterosexual - serodifferent partner)

*“I don’t talk (...) I told my aunt, and she didn’t believe me. She said I was hiding HIV, that I had caught it from my husband. To protect him, I don’t tell anyone”* (Participant 20 – cis woman - heterosexual - Serodifferent partner)

*“I ended up talking to some people about PrEP. The first question was: but why you? Aren’t you married? Don’t you live with your husband? So, I don’t want to expose him, you know”* (Participant 14 – Cis man - homosexual)

*“They either think I’m a prostitute or that I have risky sexual exposure. That I’m promiscuous, a slut all the time, or that I live with someone who has HIV”* (Participant 14 – Cis man – homosexual)

*“I work independently; I work in production. I don’t always have demand. So, it’s more flexible. There are people who work all day, sometimes spending two hours on the bus”* (Participant 07 – Cis man – homosexual)

**4.3 Decisions about PrEP use, interruption, and resumption**

*“I use condoms, but it gave me more freedom (...) I relaxed the rules in this case”* (Participant 03 – Cis man - homosexual)

*"* *One of the reasons I stopped taking PrEP was because I was in a monogamous relationship. (...) I felt comfortable and trusted the person. So, I believe it wasn't necessary, because we were both monogamous"* (Participant 03 – Cis man – homosexual)

*“I stopped using PrEP a while ago. Many people at the company were being fired (...) I didn’t want to leave my job to seek treatment because I was afraid of being seen badly”* (Participant 13 – Cis man – homosexual)

*“I stopped for a while (stopped using PrEP). I took a break. As a salesperson, I’m pressured to produce more (...) There came a point where the company was apprehensive, with a lot of people being fired. I didn’t want to leave my job to seek treatment because I was afraid of being frowned upon” (Participant 13 – Cis man – homosexual)*

**4.4 Behavioral changes associated with PrEP use**

*“I even started getting involved with people I already knew better, you know, with whom I already had a history, or something like that, but always with protection. But then I started to be more careful”* (Participant 18 – cis woman - bisexual)

*“I started to take better care of my health, I started to get tests done for things that my daily routine didn’t allow me to do, it was just work and home, I always put my health aside, I started to think more about myself, about my health”* (Participant 06 – cis woman - heterosexual - serodifferent partner)
